# Piezoelectric Energy Generators Based on Spring and Inertial Mass

**DOI:** 10.3390/ma11112163

**Published:** 2018-11-01

**Authors:** Sanghyun Yoon, Jinhwan Kim, Kyung-Ho Cho, Young-Ho Ko, Sang-Kwon Lee, Jung-hyuk Koh

**Affiliations:** 1 School of Electrical and Electronics Engineering, Chung-Ang University, Seoul 06974, Korea; moon_light90@naver.com (S.Y.); kjh2203kjh@naver.com (J.K.); 2 Agency for Defense Development, Daejeon 34186, Korea; redskin99@naver.com (K.-H.C.); kohjunghyuk@hanmail.net (Y.-H.K.); 3 Department of Physics, Chung-Ang University, Seoul 156-756, Korea; sangkwonlee@cau.ac.kr

**Keywords:** piezoelectric, energy harvester, shock absorber

## Abstract

In this study, inertial mass-based piezoelectric energy generators with and without a spring were designed and tested. This energy harvesting system is based on the shock absorber, which is widely used to protect humans or products from mechanical shock. Mechanical shock energies, which were applied to the energy absorber, were converted into electrical energies. To design the energy harvester, an inertial mass was introduced to focus the energy generating position. In addition, a spring was designed and tested to increase the energy generation time by absorbing the mechanical shock energy and releasing a decreased shock energy over a longer time. Both inertial mass and the spring are the key design parameters for energy harvesters as the piezoelectric materials, Pb(Mg_1/3_Nb_2/3_)O_3_-PbTiO_3_ piezoelectric ceramics were employed to store and convert the mechanical force into electric energy. In this research, we will discuss the design and performance of the energy generator system based on shock absorbers.

## 1. Introduction

Recently, various efforts have been made to solve the energy shortage problem owing to the depletion of fossil fuels. Nowadays energy efficient systems and small devices with low energy consumption are very important [1,2]. The piezoelectric ceramic is one of the renewable energy materials that has attracted attention [3,4,5,6]. Due to its high reliability based on low-cost processing, sustainable properties and various application such as sensing devices and converters [7,8,9,10,11], piezoelectric is a suitable material for an energy harvester [12,13]. Among the various types of energy harvesting sources [14,15,16,17,18,19], shock absorber systems have a lot of merits as a piezoelectric energy harvesting system [20]. The shock absorber systems are made from elastic materials such as plastic resins or metals with springs. In general, shock absorber systems can only dissipate shock energy as heat energy or sound energy [21,22]. However, shock energy has not been yet considered as an energy source. In addition, the spring structure can change the energy shape from an impulse shape with a short shock time to a unit shape with a long shock time. The total amount of energy cannot be changed but the applied shock time can be modulated [23]. By employing piezoelectric materials to the shock absorber to collect the wasted energy, the efficiency of the shock absorber can be increased. In this research, we have designed and tested a piezoelectric energy harvester system based on a shock absorber with an inertial mass and a spring. Piezoelectric materials can be employed in the shock absorber system to convert mechanical shock energy into electrical energy and should be designed to withstand strong mechanical energy. Since piezoelectric materials have a limited mechanical strength and brittle properties, spring-based structures can be effective structures to collect and store the mechanical energy, which is transferred to the active shock absorber system. With an appropriately designed active shock absorber [24], it can act as a damper to protect the main system as well as to harvest the applied energy. The piezoelectric energy harvester, based on the shock absorber system, consists of piezoelectric material, a spring, and a transfer plate, which can deliver a uniform force. By using a spring system, which can elongate the energy generation time with decreased power density, piezoelectric energy harvesters based on shock absorber systems can generate energy when the materials are exposed to positive or negative mechanical forces. Due to the short generation time of piezoelectric ceramics, it is important to employ a spring structure to increase the generation time and store the energy [25]. As a piezoelectric material, lead zirconate titanate (PZT) is the most popular piezoelectric material [26]. However, because of the excellent dielectric and electromechanical properties, the PbMgNbO_3_-PbTiO_3_ is the best substitute for PZT in many devices and applications [27,28]. Various studies and applications have already proceeded. However, as an energy source, there are very limited reports about the equivalent circuit for the voltage sources. A piezoelectric energy harvester can be designed as the mechanical force-dependent voltage source. In this research, we have modeled a piezoelectric energy generator as a mechanical force-dependent voltage source and tested the circuit. 

In this article, we have designed an energy generator system based on the active shock absorber system and applied it to the equivalent circuit to maximize the generated output energy.

## 2. Materials and Methods

### 2.1. 0.675PMN-0.325PT Ceramics

**Figure 1:**
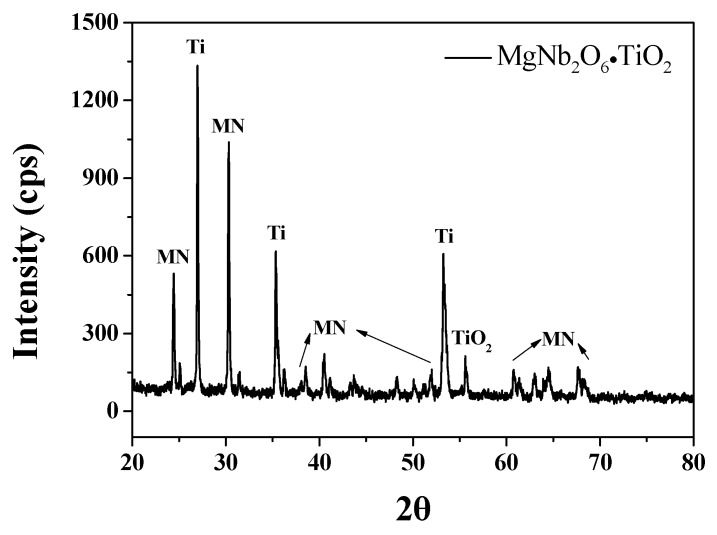
X-ray diffraction patterns for the MgNb_2_O_6_·TiO_2_ powders.

0.675PbMgNbO_3_-0.325PbTiO_3_ (PMN-PT) powder was employed to fabricate piezoelectric ceramics [29]. The powders used as the initial materials were magnesium carbonate (Sigma Aldrich, Darmstadt, Germany, 99%), niobium pentoxide (Sigma Aldrich, 99%), titanium dioxide (Sigma Aldrich, 99%), lead nitrate (Sigma Aldrich, 99%) and oxalic acid (Sigma Aldrich, 99%). The stoichiometric ratio was matched to weigh the initial materials. First, MgCO_3_ and Nb_2_O_5_ were milled together with methanol (Merck, Darmstadt, Germany, 99.9%) and zirconia balls using a high energy planetary ball mill and dried in an oven at 120 °C. After the drying process, the powder was calcined at 1100 °C for 4 h in air. The powder was mixed with weighed titanium dioxide by planetary ball milling for 6 h with 150 rpm. To prepare the MNT powder, the mixed powder was calcined for 4 h at 1100 °C, as shown in Figure 1. The lead nitrate was added in the form of a 1.5 molar aqueous solution at a ratio of 1:1 with MNT molar ratio, and further stirred for 3 h to coat lead oxalate onto the MNT particles. The powder was calcined at 750 °C for 4 h after being dried at 120 °C. A disk-type sample was made with the calcined powder and sintered at 1275 °C for 2 h. The fabricated structures of the samples were investigated by X-ray diffraction (XRD) analysis (RigakuModel D/MAX-2500V/PC, Bruker-AXS, Billerica, MA, USA). The structure was observed using field emission scanning electron microscopy (FE-SEM) (Hitachi S-4300, Carl Zeiss, Oberkochen, Germany). The frequency dependence of the dielectric constant (*ε_r_*) and dielectric loss (*tan δ*) of the samples were measured by employing an impedance analyzer from 1 kHz to 1 MHz (Agilent 4294A Precision, Agilent Technologies, Santa Clara, CA, USA).

### 2.2. Piezoelectric Energy Harvester with Spring-Based Shock Absorber

Piezoelectric energy harvesters were prepared by introducing the inertial mass-based structure with and without springs. With and without spring-based structures were prepared and compared for the energy harvesting capabilities. The mechanical impact energy was applied to the specimens by a droplet test system that dropped the mass of 240 and 720 g [30]. The distance between the droplet system and energy harvester was 40 cm. As a result, the calculated pressure was around 40 MPa for 240 g. The impedance matching circuit was prepared by employing a full-bridge rectifier to store the generated output energy. The generated open circuit voltage was measured by the oscilloscope (Agilent DSO-X2002A, Agilent Technologies, Santa Clara, CA, USA). The output voltage was applied using a 10 μF capacitor to extract the energy.

## 3. Results and Discussion

**Figure 2:**
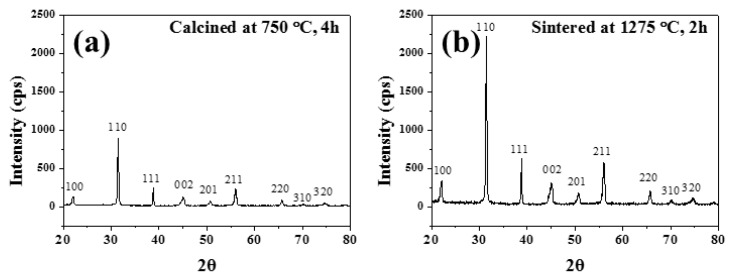
X-ray diffraction patterns for the 0.675PbMgNbO_3_-0.325PbTiO_3_ powders after calcination (**a**) and after sintering (**b**).

The X-ray diffraction (XRD) patterns of the 0.675PMN-0.325PT calcined powder and sintered ceramics are shown in Figure 2a,b. X-ray diffraction analyses were performed to determine the structural properties. As shown in the figure, there was no pyrochlore phase and it had a perovskite structure. After the calcination process, the PMN-PT powders had a perovskite structure. The peak intensities were 2.5 times increased after the sintering process without the pyrochlore phase.

**Figure 3:**
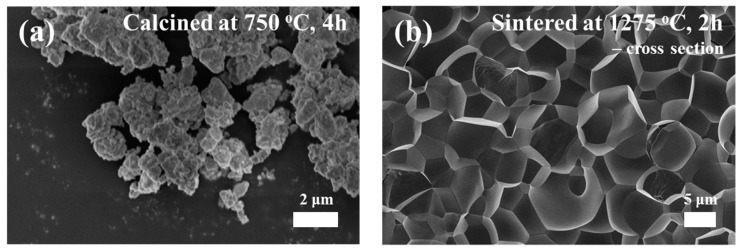
SEM images of the 0.675PbMgNbO_3_-0.325PbTiO_3_ powders after calcination (**a**) and after the sintering (**b**).

Figure 3a,b shows the FE-SEM images for the PMN-PT calcined power and sintered ceramic specimen. As shown in Figure 3a, the powders had a sub-micron particle size with uniform size. In addition, Figure 3b shows the cross-section images of the PMN-PT ceramics prepared by FE-SEM. By observing the cross-section of the PMN-PT ceramics, the grain size was estimated. The observed grain size was around 7 μm. From these FE-SEM images, it was confirmed that the grain was well grown and the sintering process was completed, without defect.

**Figure 4:**
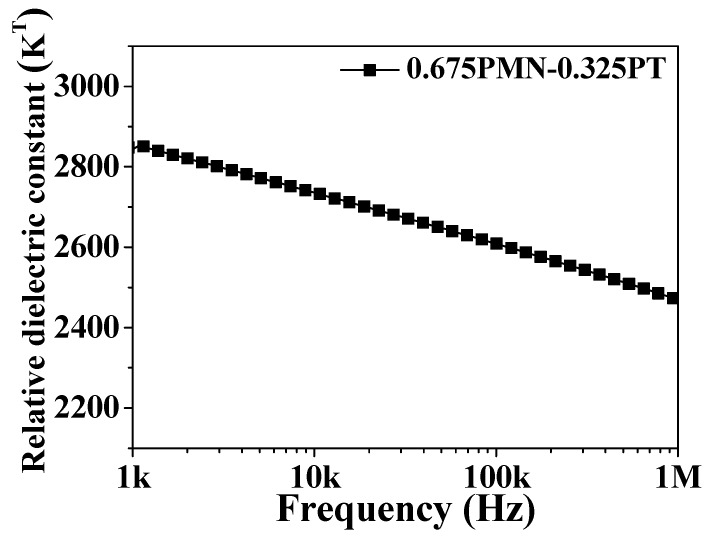
The frequency dependent relative dielectric constant of the 0.675PbMgNbO_3_-0.325PbTiO_3_.

Figure 4 displays the frequency dependent dielectric constant of 0.675PMN-0.325PT ceramics, which were employed in the shock absorber system. The dielectric constant was measured by varying the frequency from 1 kHz to 1 MHz. The dielectric constant decreased from 2847 to 2469 as the frequency increased from 1 kHz to 1 MHz. 

**Figure 5:**
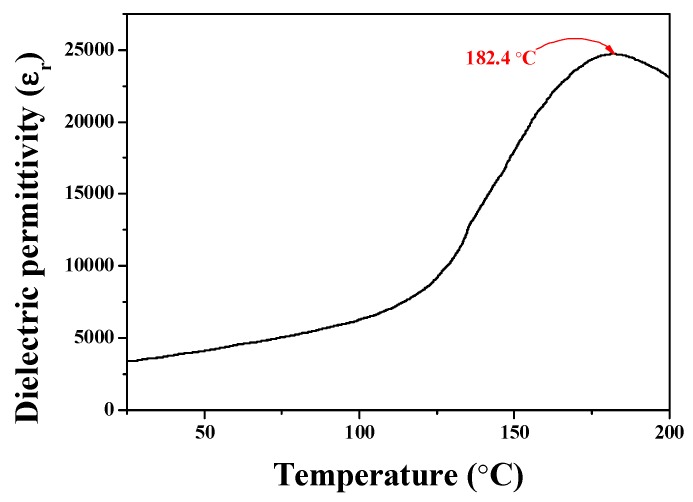
Temperature-dependent dielectric constant of the 0.675PbMgNbO_3_-0.325PbTiO_3._ ceramics and Curie temperature.

Figure 5 reveals the Curie temperatures of the PMN-PT system. As shown in the figure, the Curie temperature of the PMN-PT system was ~182.4 °C. The Curie temperature was slightly lower than that of the PZT system. As shown in Table 1, the piezoelectric charge coefficient of PMN-PT was around 530, which was slightly higher than that of the PZT system. In general, the piezoelectric charge coefficient and the Curie temperature had a trade-off relationship. Even though the Curie temperature was lower than that of PZT, the measured piezoelectric charge coefficient was slightly higher than that of PZT. Since this shock absorber system was designed to absorb energy, which has a mechanical force with an impulse shape, it is more desirable to have a higher piezoelectric charge coefficient by sacrificing the Curie temperature.

**Figure 6:**
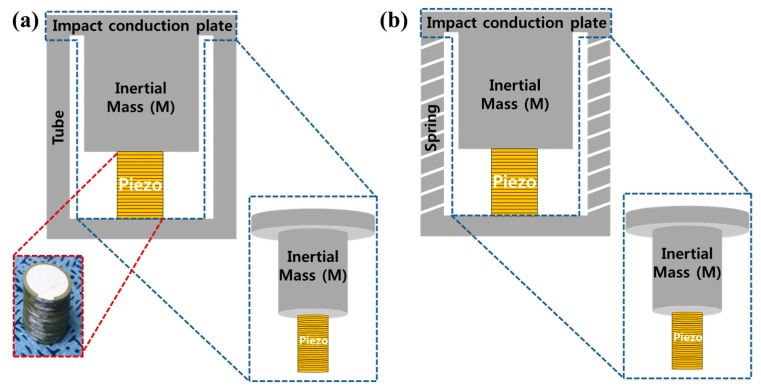
Schematic diagram and a photograph of the piezoelectric energy harvesting systems based on a shock absorber without a spring structure (**a**) and with a spring structure (**b**).

Figure 6 explains the schematic diagram of the piezoelectric energy generators based on the shock absorber system. A schematic diagram of a spring-based energy harvester system combining the piezoelectric energy harvester and an inertial mass is shown in Figure 6b. Figure 6a shows the energy harvesters without a spring structure. Spring-based energy harvester systems were made of stainless steel, in which all specifications of the spring, the inertial mass, and the impact conduction plate were prepared with the same stainless steel materials. The difference between Figure 6a and Figure 6b is the outer spring structure. The piezoelectric energy harvesters had piezoelectric ceramic plates, which were laminated to a size of 30 layers with a diameter of 0.3 mm.

**Figure 7:**
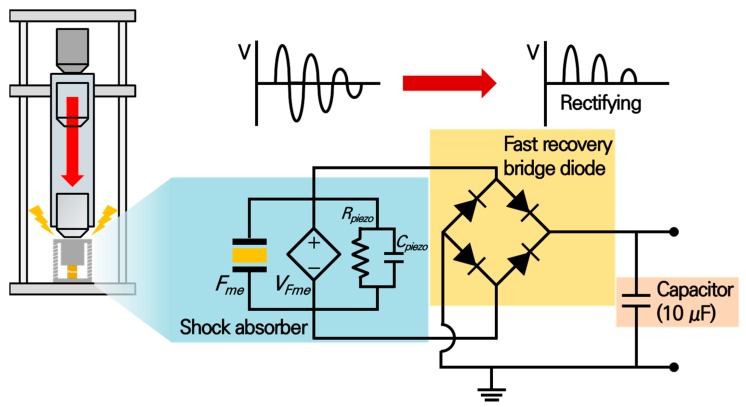
The schematic diagram of the energy storing system with a fast recovery bridge diode and shock absorber.

Usually, mechanical shock energy can be applied to the shock absorber system, the forcing time is very short and it is almost impossible to generate or collect the energy. To solve this problem, a piezoelectric energy generator was incorporated with a shock absorber system and the energy storage circuit was attached to maximize the collecting energy. Figure 7 shows the designed energy storage circuit system. A fast recovery diode-based bridge rectification system was suggested to be employed in this research. Since the voltage generation time of piezoelectric energy harvesters without a spring structure is very short, around a sub-microsecond, some amount of energy cannot be collected due to the time lost from the recovery time of the diodes. Therefore, it is very important to employ a fast diode-based full-bridge rectifying system [33]. The voltage generated by the energy harvester was stored in a 10 μF capacitor across the bridge rectification system and the generated energy was derived by measuring the voltage applied to the capacitor.

**Figure 8:**
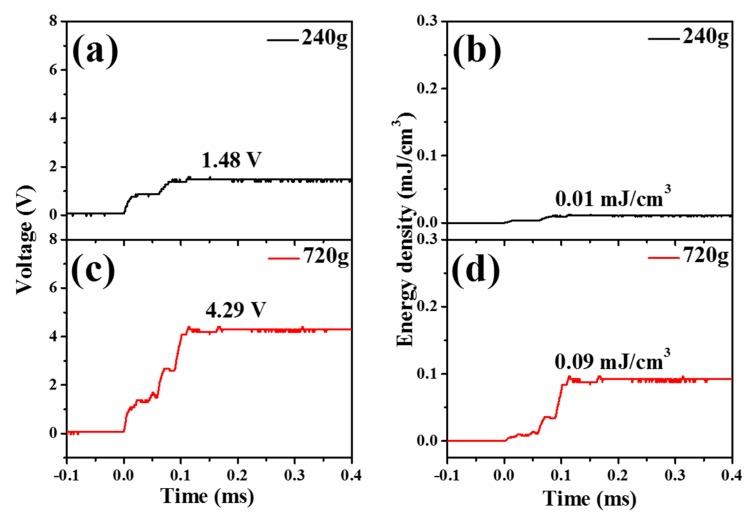
The generated output voltage and energy from piezoelectric energy harvesters without a spring structure. The energy was stored in a 10 μF capacitor. The generated output voltage of a 240 g weight (**a**), generated energy of a 240 g weight (**b**), generated output voltage of a 240 g weight (**c**), generated energy of a 720 g weight (**d**).

**Figure 9:**
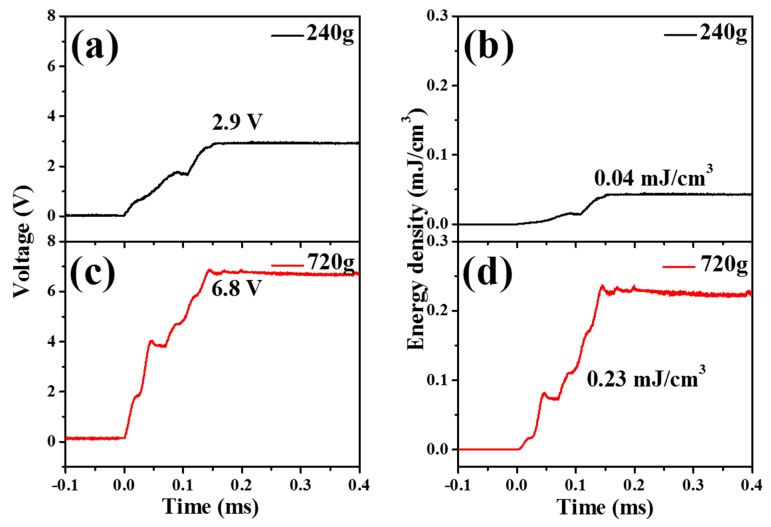
The generated output voltage and energy from piezoelectric energy harvesters with a spring structure. The energy was stored in a 10 μF capacitor. The generated output voltage of a 240 g weight (**a**), generated energy of a 240 g weight (**b**), generated output voltage of a 240 g weight (**c**), generated energy of a 720 g weight (**d**).

Figure 8 and Figure 9 show the generated voltage and output energy values from the piezoelectric energy harvesters without and with spring systems. The generated voltage was rectified through the full-bridge rectifier and then stored in the capacitor. Massed of 240 g and 720 g were dropped on the shock absorber system, which contained piezoelectric materials and an inertial mass. Voltages (1.48 and 4.29 V) were generated from the piezoelectric energy harvester without a spring structure, which corresponded to 0.01, and 0.09 mJ/cm^3^ for the droplet for 240 (Figure 8b) and 720 g (Figure 8d), respectively. The generated voltage applied to the capacitor was converted to the energy value using the following equation [34]:

(1) $$Energy\;\left(E\right)=\frac{1}{2}\;CV^{2},$$

where *C* is the capacitance value to measure the voltage and *V* is the measured voltage in the capacitor.

Figure 9a shows the generated voltage by dropping 240 g onto the piezoelectric energy harvesters with a spring structure. The piezoelectric energy harvesters had the same number of layers as piezoelectric plates. Compared with Figure 8, it can be seen that the output voltage and energy were increased by introducing the spring structure. Masses of 240 g and 720 g were dropped on the energy harvester system, which contained piezoelectric materials and an inertial mass. Voltages (2.9 and 6.8 V) were generated from the piezoelectric energy harvesters with a spring structure, which corresponded to 0.04, and 0.23 mJ/cm^3^ for the droplet for 240 (Figure 9b) and 720 g (Figure 9d), respectively.

By comparing Figure 8 and Figure 9, it seems that piezoelectric energy harvesters with a spring structure can generate more energy than that without a structure. By employing the spring-based structure, the applied force time of the piezoelectric energy harvesters can be elongated. By considering the spring-based structure and the fast recovery based rectifying circuit system, the efficiency of piezoelectric energy harvesters can be improved. The generated output energy was improved around 2.5 times. It seems that it is important to design the inertial mass-based structure to concentrate the applied energy to the piezoelectric material and design the spring-based structure to elongate the applied time to the piezoelectric material to increase energy generation. 

## 4. Conclusions

In this paper, we have designed and analyzed piezoelectric energy generators based on a shock absorber with and without a spring structure. The designed piezoelectric energy harvesters can absorb and convert the applied mechanical energy into electrical energy. A rectifier circuit system employing a fast recovery diode was designed to more efficiently store the electrical energy. A spring system was designed and employed to maintain the mechanical energy for a longer time. In our research, we found that piezoelectric energy harvesters with an inertial mass and spring structure and rectifying system with a fast recovery diode-based full bridge system can collect and store the electrical energy more efficiently. Especially, by introducing the spring-based system, the collected energy was increased by 2.5 times.

## Figures and Tables

**Table 1:** Characteristics of the prepared PMN-PT.

Materials	Piezoelectric Constant (*d_33_*)	Dielectric Constant (*ε_r_*)	Curie Temperature (*T_c_*)	Density (g/cm^3^)	Planar Coupling Factor (*K_p_*)	Mechanical Quality Factor (*Q_m_*)
Pb(Zr_0.52_Ti_0.48_)O_3_ [31,32]	223	1240	377	7.55	52.9	-
0.675PMN-0.325PT	530	2847	182.4	7.532	78.1	65.3
